# Quantitative evaluation of the molecular marker using droplet digital PCR

**DOI:** 10.5808/GI.2020.18.1.e4

**Published:** 2020-03-31

**Authors:** Wonseok Shin, Haneul Kim, Dong-Yep Oh, Dong Hee Kim, Kyudong Han

**Affiliations:** 1 Department of Nanobiomedical Science & BK21 PLUS NBM Global Research Center for Regenerative Medicine, Dankook University, Cheonan 31116, Korea; 2 Center for Bio-Medical Engineering Core Facility, Dankook University, Cheonan 31116, Korea; 3 Livestock Research Institute, Yeongju 36052, Korea; 4 Department of Anesthesiology and Pain Management, Dankook University College of Medicine, Cheonan 31116, Korea

**Keywords:** droplet digital PCR, Hanwoo-specific marker, structure variation

## Abstract

Transposable elements (TEs) constitute approximately half of Bovine genome. They can be a powerful species-specific marker without regression mutations by the structure variation (SV) at the time of genomic evolution. In a previous study, we identified the Hanwoo-specific SV that was generated by a TE–association deletion event using traditional PCR method and Sanger sequencing validation. It could be used as a molecular marker to distinguish different cattle breeds (i.e., Hanwoo vs. Holstein). However, PCR is defective with various final copy quantifications from every sample. Thus, we applied to the droplet digital PCR (ddPCR) platform for accurate quantitative detection of the Hanwoo-specific SV. Although samples have low allele frequency variation within Hanwoo population, ddPCR could perform high sensitive detection with absolute quantification. We aimed to use ddPCR for more accurate quantification than PCR. We suggest that the ddPCR platform is applicable for the quantitative evaluation of molecular markers.

## Introduction

Hanwoo (*Bos taurus coreanae*) is a domesticated mammal that has been used for agriculture and transportation since 5,000 years ago [1]. As the Korea economy developed in 1960, it began to provide as one of food resources [2]. In particular, Hanwoo is consumed more beef than other cattle breeds in Korea [3]. This consumption pattern has led to the emergence of research on the development of molecular makers that distinguish between Hanwoo and other cattle breeds [4-6].

In a recent study, they investigated Hanwoo-specific structural variation (SV) using BreakDancer program (ver 1.1) to distinguish between Hanwoo and Holstein [7]. The SVs typically included insertion, deletion, inversion, translocation, and copy-number variation [8-10]. SVs could affect much greater genomic function and gene expression than single nucleotide variants [11]. In this respect, the previous study focused on transposable element (TE)-mediated deletion events. Thus, Park et al. [7] identified an authentic Hanwoo-specific deletion locus that was confirmed by PCR and Sanger sequencing. It can be utilized to distinguish between Hanwoo and Holstein species. However, PCR has several defects in detecting DNA amplification. For example, contaminated sample including trace amounts of DNA might lead to misleading outputs [12]. In addition, the specificity of the PCR product could be affected by non-specific binding of the primers to other similar sequences on the template DNA [12]. Complementing these drawbacks, the quantitative PCR (qPCR) could estimate target DNA quantity using either a fluorescent dye (e.g., SYBR Green) that non-specifically intercalates with double-stranded DNA (dsDNA) or TaqMan probe assay. Nevertheless, most qPCR methods rely on the precise number of copies compensated by calibrator, assuming no loss of calibrator molecules during the all experimental steps [13]. However, errors can occur at several levels [14,15]. In addition, the qPCR has the following disadvantages. (1) The accuracy of qPCR depends on proper experimental design based on well-established reference genes. (2) For absolute quantification, you should create a standard curve for data normalization based on changes in the transcription level of the reference gene [16].

The droplet digital PCR (ddPCR) is one of next-generation technologies for absolute quantification of nucleic acids [17]. It counted the fluorescent PCR-positive and PCR-negative droplets to calculate target DNA concentration and thus absolute quantification was directly estimated as the exact number of copies without the aid of calibration curve [15]. Currently, seven commercial digital PCR systems (Thermo Fisher Quantstudio 3D, Fluidigm BioMark qdPCR 37K, Formulatrix Constellation, JN Medsys Clarity, Bio-Rad QX200, Raindance Raindrop plus, and Stilla Naica) are available [18]. Among them, the Stilla Naica System for Crystal Digital PCR [19] has a predominant feature of step emulsion generators. It is not necessary to do the flow of oil by developing the Sapphire chip, which development has simplified the operation and reduced potential contamination.

This study uses ddPCR, the Stilla Naica System for Crystal Digital PCR, to overcome the limitations of PCR and to accurately evaluate the Hanwoo-specific SV locus that was identified in the previous study [7]. We suggest that the ddPCR platform can be used as a quantitatively and numerically sensitive method with molecular markers.

## Methods

**Fig. 1.**
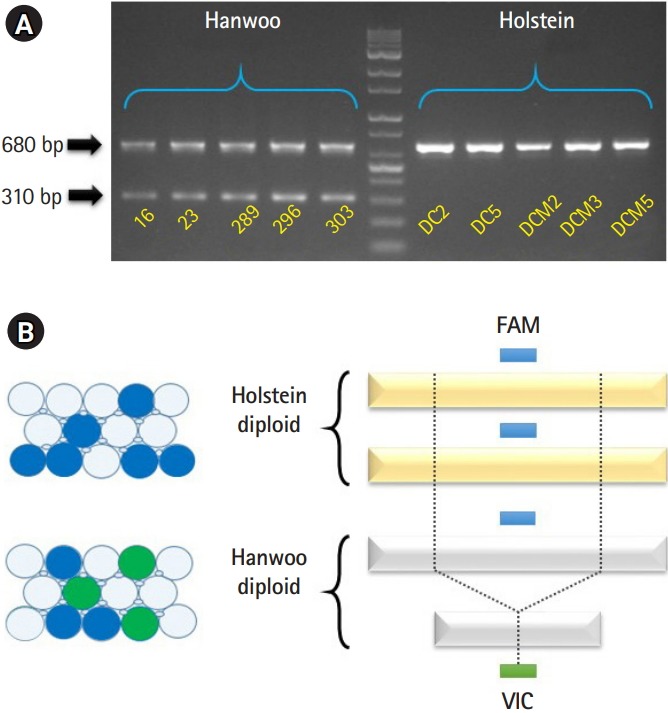
Structural variation of the Hanwoo and Holstein genomes. (A) Polymorphic pattern of the Del_96 locus in the Hanwoo and Holstein cattle samples [7]. Gel chromatography showed that five Hanwoo samples (left panel) contained heterozygous alleles (680 bp and 310 bp) but five Holstein samples (right panel) had no the deleted allele (680 bp). (B) To analyze absolute quantification using droplet digital PCR assay, the FAM probe (blue box) was designed to detect all cattle genome (positive control). The VIC probe (green box) was designed in boundary of Hanwoo-specific deletion (Del_96).

The five brown Hanwoo DNAs and five Holstein DNAs were extracted from blood samples using the DNeasy Blood & Tissue kit according to the manufacture’s instruction (Qiagen, Hilden, Germany). All research protocols and animal experiments in this study were reviewed and approved by the Institutional Animal Care and Use Committee (IACUC) in Gyeongsangbuk-do, Republic of Korea (Gyeongbuk IACUC-87). Next, we confirmed the PCR amplicon pattern of the “Del_96” locus [7] from all samples by PCR. The Hanwoo samples showed a polymorphic pattern of PCR products (680 bp/310 bp) generated by TE-association deletion event. However, Holstein samples contained no the deleted allele, so only PCR products of 680 bp are observed (Fig. 1A).

To more accurately detect the Hanwoo-specific SV, we have applied the “Del_96” locus [7] to the ddPCR platform (Stilla Technologies, Villejuif, France). The FAM primer set and FAM probe (Thermo Fisher Scientific, Waltham, MA , USA) were used for the detection of both Hanwoo and Holstein genomes. The VIC primer set and VIC probe (Thermo Fisher Scientific) were designed at the boundary of Hanwoo-specific deletion (Fig. 1B). Thus, we designed that the FAM primer set and FAM probe were detected in all cattle DNAs (positive control). The VIC primer set and VIC probe were designed to detect fluorescence only in the Hanwoo cattle. We followed the manufacturer’s instructions for experimenting with the ddPCR platform. Prior to the experiment, we confirmed the quantification of Hanwoo and Holstein DNAs using Qubit 4.0 Fluorometer (Thermo Fisher Scientific) with 1× dsDNA HS (high-sensitivity) assay kit (Thermo Fisher Scientific) for dsDNA measurement.

The ddPCR reaction mixture (25 µL) contained 12.5 µL of PerfeCta qPCR ToughMix UNG 2× (Quanta Biosciences, Gaithersburg, MD, USA), 2.5 µL of 100 nM of Fluorescein (VWR International, West Chester, PA, USA), 1.25 µL of primer set/VIC probe (final concentration of 900 nM/250 nM, respectively), 50 ng DNA, and nuclease-free water up to 25 µL. The reaction mixtures were loaded into wells of Sapphire chip (Stilla Technologies), respectively. Then, the chips are placed into the Naica Geode equipment and we launched the combined partitioning and thermocycling program. The ddPCR condition was initial denaturation step of 3 min at 95℃, followed by 45 cycles of 95℃ for 10 s and 60℃ for 15 s, with a release step for 33 min to down temperature and pressure. 20,000 to 30,000 droplets are created from each sample. At the end of template amplification from the separated droplets, the chips were transferred to the Naica Prism3 reader. Finally, extracted fluorescence values for each droplet were analyzed using the Crystal Miner software (Stilla Technologies). Thresholds were set using the automation tools available in the Crystal Miner software.

## Results and Discussion

Hanwoo-specific deletion locus (Del_96 region) was found in a previous study by comparing the cattle genomes with whole-genome sequencing data and proved by PCR and Sanger sequencing methods [7]. It has been reported that the Del_96 region occurred through nonallelic homologous end-joining between LINE (BovB) and unique sequence only in the Hanwoo genome [7]. It can be used as a powerful marker for distinguishing Hanwoo and Holstein (Fig. 1A). Even though validation experiment based on PCR method used in their study are easy to perform at small sample size, the PCR method can be affected by nonspecific binding of primer set to similar sequences on the gDNA [12]. To overcome the shortcomings of the PCR method and apply next-generation technology, we try to verify the Hanwoo-specific deletion region by a ddPCR assay.

To perform the ddPCR assay, we designed two probes (Supplementary Table 1). One designed a positive control probe (FAM dye; blue) to detect all cattle genomes, and the other to a Hanwoo-specific deletion boundary site (VIC dye; green) (Fig. 1B). DNA templates from five Hanwoo and five Holstein blood samples were conducted to the ddPCR assay with designed primer/probe sets. The extracted DNAs should be assessed for accurate quantification using a UV spectrophotometer (NanoDrop, Thermo Fisher Scientific) and an intercalating reagent reaction with the dsDNA (Qubit assay). In particular, it is important to quantify dsDNA because dsDNA of total DNA actually reacts in the ddPCR assay (Table 1) [20].

**Fig. 2.**
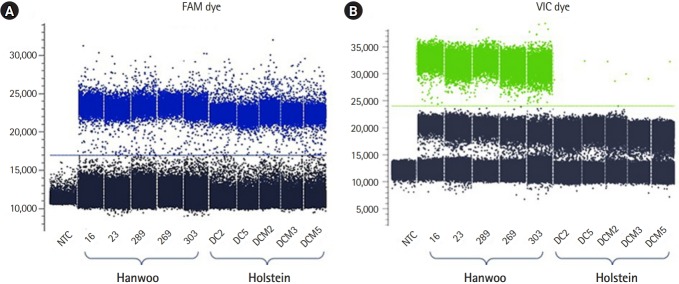
1D-Dot plot display of mono-color droplet fluorescence intensity. The dots indicate each droplet that was detected by FAM (left plot) and VIC (right plot) dyes using the droplet digital PCR assay. (A) The X- and Y-axis indicate the name of each sample and the number of droplets with positive fluorescence intensity with the FAM probe (blue color), respectively. (B) The X- and Y-axis indicate the name of each sample and the number of droplets with positive fluorescence intensity with the VIC probe (green color).

**Fig. 3.**
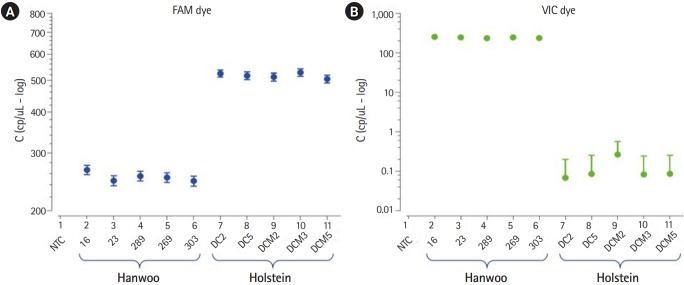
Absolute copy number comparison in Del_96 region between Hanwoo and Holstein samples. The concentration graph indicates sample number on the X-axis and log scale bar (copy/μL) on the Y-axis. (A) The FAM fluorescence was detected in all samples. The absolute copy number of Hanwoo samples were approximately two times less than that of Holstein samples. (B) The VIC fluorescence was only detected strongly in Hanwoo samples.

The Stilla Naica system yields between 20,000 and 30,000 analyzable droplets. In this study, we generate an average approximately 22,392 of droplets using the Stilla Naica system (Table 2). Thus, there are enough droplets to analyze the absolute copy number. As shown in Fig. 2, FAM dye was detected in all cattle genomes and VIC dye showed significant detection only in the Hanwoo samples. It suggests that all Hanwoo genomes contain the specific deletion sequence (Del_96 region). Signals of VIC dye were detected on average 243 Channel concentration (copy/μL) in the Hanwoo samples. However, an average of 0.12 Channel concentration (copy/μL) VIC dye signals, which were very few and insignificant droplets, were also detected in the Holstein samples. In the previous study, the Del_96 region deleted from the Hanwoo genome was reported to occur in one of the transposable elements, the BovB element region. At present, the cattle reference genome (bosTau9 version) has not well annotated the segmental duplication region and TE positions. Therefore, it is important to consider that VIC probe designed in the TE region can detect non-specific signals on sequences with high similarity. In addition, the signals obtained from these droplets could be recognized as false signals due to the abnormally high fluorescence intensity measured in ddPCR assay [21,22]. Nevertheless, the difference in the average number of VIC dyes detected between the Hanwoo and Holstein samples was statistically sufficient to distinguish them. Our results show that the ddPCR assay is very appropriate to distinguish between Hanwoo and Holstein cattle. On the other hand, the signals of the FAM dye were detected on average 253.5 Channel concentration (copy/μL) in the Hanwoo samples and an average of 516.7 Channel concentration (copy/μL) FAM dye signals were also detected in the Holstein samples (Fig. 3). As shown in Fig. 1B, we designed a FAM probe/primer set for the sequence that exist within Hanwoo-specific deletion region. Thus, the copy numbers that were detected by FAM dye signal were observed two times more in Holstein samples than Hanwoo samples.

In ddPCR assay, DNA is divided into numerous wells or droplets, and the concentration of target region is absolute quantified using Poisson statistics [23,24]. The ddPCR assay can be quantified with high accuracy in counting single molecules and analyzing a small number of copies of a particular population [25,26]. However, consumable and equipment cost for ddPCR are still expensive compared to those of qPCR.

For the ddPCR technology, accurate quantification of absolute copy number is a key feature. In the near future, by applying species-identifying makers to ddPCR, it has significant potential as a platform for species identification at large sample sizes. Taken together, we propose that ddPCR is suitable as a platform for verifying species-specific markers.

## Figures and Tables

**Table 1.** Cattle gDNA quality control and dsDNA concentration

Sample name	Microvolume spectrometer			Qubit fluorescence 4.0 dsDNA concentration (ng/μL)
	Concentration (ng/μL)	A260/A280	A260/A230	
Hanwoo_#16	33.2	1.92	1.1	30.5
Hanwoo_#23	35.1	1.83	1.26	16.7
Hanwoo_#289	35.1	2.01	1.8	41.1
Hanwoo_#296	23.8	1.7	1.74	22.6
Hanwoo_#303	18.8	1.78	1.76	16
Holstein_DC2	36.6	1.87	1.8	33.4
Holstein_DC5	85.5	1.91	1.64	37.5
Holstein_DCM2	34	1.93	1.18	28
Holstein_DCM3	29.2	1.91	1.67	28.2
Holstein_DCM5	25.5	1.63	1.38	25.8

gDNA, genomic DNA; dsDNA, double-stranded DNA.

**Table 2.** Statistical result of the ddPCR assay

Sample name	Total No. of droplets	FAM dye			VIC dye		
		Channel concentration (copy/μL)	No. of positive droplets	p-value	Channel concentration (copy/μL)	No. of positive droplets	p-value
NTC	23,549	0	0	N/A	0	0	N/A
Hanwoo_#16	23,782	266.3	3,436	0.0335	253.2	3,279	0.0343
Hanwoo_#23	24,123	247	3,250	0.0344	244.8	3,223	0.0346
Hanwoo_#289	23,601	255	3,275	0.0343	234.2	3,026	0.0357
Hanwoo_#296	23,568	252.6	3,242	0.0345	245	3,152	0.0349
Hanwoo_#303	22,839	246.5	3,071	0.0354	236.2	2,952	0.0361
Holstein_DC2	25,068	524.1	6,628	0.0242	0.07	1	1.96
Holstein_DC5	20,006	516	5,220	0.0272	0.09	1	1.96
Holstein_DCM2	19,341	511.3	5,007	0.0278	0.26	3	1.132
Holstein_DCM3	20,577	527.9	5,474	0.0266	0.08	1	1.96
Holstein_DCM5	19,863	504.2	5,081	0.0276	0.09	1	1.96

ddPCR, droplet digital PCR; N/A, not available.

## Supplementary Materials

Supplementary Table 1. Information of ddPCR primer and probe sequences
