# Supplementary material for: Quantitative evaluation of the molecular marker using droplet digital PCR
Source: Genomics Inform. 2020 Mar 31;18(1):e4. doi: 10.5808/GI.2020.18.1.e4 (PMC7120350; doi:10.5808/GI.2020.18.1.e4)
Supplement: Supplementary Table 1. — Information of ddPCR primer and probe sequences [file gi-2020-18-1-e4-supple1.docx]

| **Supplementary Table S1. Information of ddPCR primer and probe sequences** | |
| --- | --- |
| **Name** | **Sequence** |
|  |  |
| Deletion region_Primer-FW | AGTTCATGTCCACTGCATTGGT |
| Deletion region_Primer-RV | GGAGAAGAGAGCATCAGCAGATG |
| Deletion region_Probe | **/FAM/**ATGCCATCAGCCATC**/MGB/** |
| Deletion boundary_Primer-FW | TTCATATATTTTGTGATTGACTGTCCTATC |
| Deletion boundary_Primer-RV | TGTGGTGCTGGACAAGATTCC |
| Deletion boundary_Probe | **/VIC/**CTTGCTTTTCCCTCCTT**/MGB/** |
